# Supplementary material for: Bone Health in Patients with Rheumatoid Arthritis in Bahrain
Source: Medicina (Kaunas). 2024 Dec 18;60(12):2078. doi: 10.3390/medicina60122078 (PMC11678244; doi:10.3390/medicina60122078)
Supplement: Supplementary file 1 [file medicina-60-02078-s001.zip › Supplementary Material Table S1.docx]

**Supplementary Material Table S1. Data associated with Vitamin D status.**

| **Characteristic** | **Vitamin D** | | **Chi-Square** | **P-Value** |
| --- | --- | --- | --- | --- |
|  | **Non optimal**  **n (%)** | **Optimal**  **n (%)** |  |  |
| **Gender** |  |  |  |  |
| Male | 144 (67.3) | 70 (32.7) | 1.115 | 0.291 |
| Female | 1636 (63.7) | 933 (36.3) |  |  |
| **Postmenopausal** |  |  |  |  |
| 56-< 60 Years | 306 (66.1) | 157 (33.9) | 8.912 | 0.012 |
| 60-<75 Years | 742 (60.9) | 477 (39.1) |  |  |
| =>75 Years | 172 (69.6) | 75 (30.4) |  |  |
| **DMT2** |  |  |  |  |
| Yes | 748 (65.9) | 387 (34.1) | 3.015 | 0.082 |
| No | 1035 (62.7) | 616 (37.3) |  |  |
| **Uric acid** |  |  |  |  |
| Normal | 989 (65.2) | 529 (34.8) | 0.503 | 0.478 |
| High | 392 (63.5) | 225 (36.5) |  |  |
| **Ca breast** |  |  |  |  |
| Yes | 173 (70.3) | 73 (29.7) | 4.687 | 0.030 |
| No | 1610 (63.4) | 930 (36.6) |  |  |
