# Supplementary material for: Bone Health in Patients with Rheumatoid Arthritis in Bahrain
Source: Medicina (Kaunas). 2024 Dec 18;60(12):2078. doi: 10.3390/medicina60122078 (PMC11678244; doi:10.3390/medicina60122078)
Supplement: Supplementary file 1 [file medicina-60-02078-s001.zip › Supplementary Material Table S2.docx]

**Supplementary Material Table S2. Data associated with Body Mass Index (BMI).**

| **Characteristic** | **BMI** | | | | P-Value |
| --- | --- | --- | --- | --- | --- |
|  | Underweight  n (%) | Normal  n (%) | Overweight  n (%) | Obesity  n (%) |  |
| **DMT2** |  |  |  |  |  |
| Yes | 15 (0.8) | 210 (11.5) | 531 (29.0) | 1076 (58.7) | < 0.001 |
| No | 73 (2.8) | 538 (20.3) | 828 (31.3) | 1209 (45.7) |  |
| **Ca breast** |  |  |  |  |  |
| Yes | 5 (0.8) | 86 (13.0) | 196 (29.6 | 375 (56.6) | < 0.001 |
| No | 83 (2.2) | 662 (17.3) | 1163 (30.5) | 1910 (50.0) |  |
| **BMD** |  |  |  |  |  |
| Normal | 11 (0.9) | 135 (10.6) | 332 (26.2) | 790 (62.3) | < 0.001 |
| Abnormal | 65 (2.7) | 484 (20.5) | 764 (32.3) | 1052 (44.5) |  |
| **Uric acid** |  |  |  |  |  |
| Normal | 29 (1.2) | 396 (16.9) | 746 (31.8) | 1178 (50.1) | < 0.001 |
| High | 7 (0.7) | 94 (9.8) | 267 (27.8) | 591 (61.6) |  |

BMD; bone mineral density. BMI; body mass index. DMT2; type 2 diabetes mellitus.
